# Circadian profiles of free plasma metanephrines in anorexia nervosa and constitutional thinness

**DOI:** 10.1007/s12020-026-04648-x

**Published:** 2026-06-08

**Authors:** Natacha Germain, Sandra Doua, Nadia Boutahar, Aurelia Gay, Catherine Massoubre, Bruno Estour, Etienne Challet, Bogdan Galusca

**Affiliations:** 1 TAPE research group, Jean Monnet University, Saint-Etienne, France; 2 Endocrinology Department, University Hospital of Saint-Etienne, Saint-Etienne, France; 3 Eating Disorder Reference Center, University hospital of Saint-Etienne, Saint-Etienne, France; 4 Biochemistry Department, University Hospital of Saint-Etienne, Saint-Etienne, France; 5 Psychiatry Department, University hospital of Saint-Etienne, Saint-Etienne, France; 6 Institute of Cellular and Integrative Neurosciences, Centre National de la Recherche Scientifique (CNRS), University of Strasbourg, Strasbourg, France

**Keywords:** metanephrines, catecholamines, 3-methoxytyramine, Dopamine, anorexia nervosa, constitutional thinness, Liquid Chromatography coupled to tandem Mass Spectrometry (LC-MS/MS), severity markers, resistance to weight gain, circadian profile

## Abstract

**Purpose:**

Catecholamine metabolism contributes to energy homeostasis and may differ in low-weight conditions of various origins. Free plasma metanephrines, 3-methoxytyramine (3-MT), normetanephrine (NM), and metanephrine (M), are stable end-products of dopamine, noradrenaline, and adrenaline metabolism, respectively, with potential circadian rhythmicity. However, their 24-hour profiles remain unknown in anorexia nervosa (AN) and constitutional thinness (CT).

**Methods:**

This exploratory study aimed to assess 24-hour variations of plasma free metanephrines in women with acute AN (*n* = 10), recovering AN (AN-Rec, *n* = 11), CT (*n* = 10), and healthy controls (*n* = 10), and to explore their association with biomarkers of undernutrition severity and weight regulation. Participants underwent 24-hour in-patient evaluation, including standardized meals and activity, and twelve time-point plasma sampling. Free metanephrines were measured by LC-MS/MS. 24-hour rhythmicity was analyzed using cosinor regression.

**Results:**

AN and CT showed higher mean 24-hour levels for 3-MT, NM, and M than controls, with variable patterns depending on metabolites. AN-Rec displayed higher 3-MT levels than controls only. Cosinor analysis revealed a significant 24-hour variation consistent with a circadian pattern for 3-MT with an acrophase around 13:00 in AN, AN-Rec, and CT. NM showed a circadian pattern in AN, CT, and controls. Mean 3-MT, NM and M correlated negatively with BMI and IGF-1, and positively with ALT and vitamin B12.

**Conclusion:**

Midday elevations of free plasma metanephrines may reflect metabolic stress and hepatic alterations in AN. Their increase may may reflect distinct underlying physiological mechanisms in both AN and CT. Further studies are needed to confirm these exploratory findings.

**Supplementary Information:**

The online version contains supplementary material available at 10.1007/s12020-026-04648-x.

## Introduction

**Anorexia nervosa (AN)** is an eating disorder characterized by severe voluntary food restriction, resulting in significant undernutrition. This condition triggers a set of adaptive endocrine and metabolic responses aimed at survival, including reduced resting energy expenditure, suppressed triiodothyronine (T3), and inhibition of the gonadotropic axis. Additionally, patients often display hypercortisolism and elevated levels of growth hormone (GH) and catecholamines [1], which help counteract the chronic hypoglycemia commonly observed in AN [2]. While these adaptations reflect the severity of the disease [1], they may also contribute to serious medical complications and, in extreme cases, sudden death [3].

In contrast, **constitutional thinness (CT)** refers to individuals with a persistently low body mass index (BMI), similar to AN, but without any signs of nutritional deficiency, psychiatric disease, or hormonal adaptation. Importantly, CT is also characterized by a paradoxical resistance to weight gain [4, 5]. Several mechanisms have been proposed to explain this phenotype, including increased brown adipose tissue (BAT) activity, which is modulated by catecholamine signaling [6]. Therefore, despite their similar BMI, AN and CT clearly differ in nutritional and hormonal profiles, raising the question of whether shared or distinct biological mechanisms underlie their persistent low weight.

Catecholamines such as dopamine, norepinephrine, and epinephrine are central neurotransmitters and hormones involved in motor function, reward signaling, endocrine regulation, and cardiovascular control [7]. These molecules are synthesized from phenylalanine and tyrosine, mainly in adrenal chromaffin cells and postganglionic sympathetic fibers [8, 9]. Notably, the gastrointestinal tract also contributes significantly to dopamine production, accounting for up to 45% of total body synthesis [10]. Each catecholamine is preferentially produced depending on the enzymatic profile of the neuron or neuroendocrine cell. Once released into the bloodstream, catecholamines are rapidly degraded. Therefore, their **more stable methoxylated metabolites**, including 3-methoxytyramine (3-MT), normetanephrine (NM), and metanephrine (M), are commonly measured to assess catecholaminergic activity [11]. Far from being inert, these metabolites, classified as **trace amines**, interact with **trace amine-associated receptor 1 (TAAR1**) [12, 13], exerting neuromodulatory and metabolic effects. These include **inhibition of food intake**,** reduced glycaemia**,** and enhanced energy expenditure**, potentially contributing to weight loss [14].

However, studies evaluating catecholamine metabolism in AN have yielded conflicting results. Early investigations focused on urinary or cerebrospinal fluid catecholamine metabolites showed inconsistent findings [15–18]. One study suggested circadian fluctuations of urinary metabolites [19], and another reported elevated plasma metanephrines in severe cases of AN [1]. In contrast, to date, no study has investigated **plasma levels of free metanephrines**, the biologically active circulating fraction, in either AN or CT.

**The present study was designed to address this gap.** We aimed to evaluate the **24-hour circadian profile of plasma free metanephrines** (3-MT, NM, and M) in four well-characterized groups of women: patients with acute AN (AN), patients with ongoing nutritional recovery from AN (AN-Rec), individuals with constitutional thinness (CT), and healthy controls. Additionally, we explored correlations between plasma metanephrine levels and key biological, nutritional, and psychometric parameters to assess their potential as biomarkers of disease severity and resistance to weight gain.

## Methods

### Study design, ethics and Participants

This was an exploratory, observational study designed to assess 24-hour patterns of free plasma metanephrines in four groups of women with different low-weight conditions or healthy status. The study was conducted in accordance with the ethical standards of the Declaration of Helsinki and was approved by the local Research Ethics Committee (IORG0007394). Written informed consent was obtained from all participants prior to inclusion.

**Forty-one women** were included and stratified into four groups:

- **Acute Anorexia nervosa (AN**,***n*** = **10)**: Patients were recruited during hospitalization for active restrictive-type anorexia nervosa, according to DSM-5 criteria [20]. All participants had secondary amenorrhea for more than 6 months and were not using hormonal contraception.
- **Ongoing recovery Anorexia nervosa (AN-Rec**,***n*** = **11)**: Patients undergoing nutritional and psychiatric follow-up for at least 6 months, with ≥ 2 BMI points gained since admission, were included.
- **Constitutional thinness (CT**,***n*** = **10)**: Participants met WHO criteria for Grade II–III thinness (BMI < 17.5 kg/m²), with preserved menstruation and spontaneous desire for weight gain, and no current or past eating disorder [20, 21].
- **Healthy controls (Controls**,***n*** = **10)**: Normal-weight (BMI 18.5–25 kg/m²) women with regular menstruation and no history of eating disorders (DSM-IV or DSM-5 criteria [20, 21]) or chronic disease were included [4].

None of the participants had any documented metabolic, psychiatric, or chronic illness. No substance use (alcohol, tobacco, illicit drugs) or medications, supplements, or hormonal contraceptives were allowed during the study.

### Sample size and study design considerations

This was an exploratory study, and no a priori sample size calculation was performed, due to the lack of existing data on 24-hour free plasma metanephrine levels in anorexia nervosa or constitutional thinness. The inclusion of approximately 10 to 11 participants per group is consistent with prior physiological studies in these rare phenotypes [4–6], and reflects the feasibility constraints associated with 24-hour in-patient protocols involving repeated sampling.

While modest, this sample size allowed for rich intra-individual data (12 time points per subject), enhancing the capacity for 24-hour modeling (cosinor regression). The present investigation is thus positioned as a hypothesis-generating study, aiming to identify preliminary biological signals for future research.

### Anthropometric evaluation

Body weight and height were measured, and BMI was calculated accordingly. Chronological age was recorded at the time of study.

### Body composition and metabolism evaluation

Total body fat mass (FM) and fat-free mass (FFM) were assessed by dual-energy X-ray absorptiometry (Lunar DPX-L; Lunar Corporation, Madison, WI; coefficient of variation < 1%) [22]. Resting energy expenditure (REE) was measured after a 12-hour overnight fast in the supine position using indirect calorimetry with a ventilated canopy system (Quark RMR, COSMED, Italy) [23].

### Psychometric Assessment

Participants completed three validated psychometric questionnaires evaluating eating-related attitudes and behaviors : the Dutch Eating Behavior Questionnaire (DEBQ) [24], the Eating Disorder Inventory-2 (EDI-2) [25], and the Eating Disorders Examination (EDE) [26].

### Sampling Protocol and Biochemical Assays

Participants were hospitalized for a 24-hour standardized protocol including: (1) Controlled sedentary activity, (2) Fixed meal times: breakfast (08:15, 400 kcal), lunch (12:15, 800 kcal), dinner (19:15, 800 kcal), and (3) No additional snacks or physical activity.

**Twelve blood samples (5 mL each)** were collected at the following time points: 04:00, 07:00, 08:00, 09:00, 10:00, 12:00, 13:00, 14:00, 16:00, 19:00, 20:00, and 24:00. After immediate centrifugation, plasma was aliquoted and stored at − 80 °C until analysis. Blood samples were collected at 12 predefined time points over a 24-hour period to obtain a sufficiently resolved nycthemeral profile while maintaining feasibility in a short in-patient protocol. This approach is consistent with our previous standardized hormonal profiling procedures and is justified by the circadian regulation of endocrine and metabolic variables. Nighttime sampling (including 04:00) was facilitated by an indwelling venous catheter, minimizing repeated venipunctures and reducing sleep disturbance as much as possible.

**Free plasma metanephrines** (3-methoxytyramine, normetanephrine, and metanephrine) were assayed using a validated LC-MS/MS method with the ClinMass^®^ assay kit (RECIPE Chemicals + Instruments GmbH, Munich, Germany). Sample preparation involved 150 µL plasma and D4-3-methoxytyramine internal standard, deposited in 96-well plates (Waters). Analysis was performed with a Xevo TQ-S micro mass spectrometer (Waters), using multiple reaction monitoring (MRM): m/z 151→91 (3-MT) and m/z 155→95 (internal standard) [27]. The 3-methoxytyramine method intra- and inter-assay precisions were determined with samples in two different concentrations: Low Level (LL) and High Level (HL). For intra-assay CV: LL (5.2%), HL (3.4%); for inter-assay CV : LL (5%), HL (2.3%).

**For additional parameters**, morning fasting (08:00) venous samples were used to measure Hemoglobin, white blood cells, platelets, electrolytes, glucose, vitamin B12, vitamin B9, liver enzymes (ALT, AST, GGT, ALP), albumin, free T3, IGF-1, estradiol, LH, and FSH. Selected parameters (leptin, GH, ACTH, cortisol, prolactin, CrossLaps, osteocalcin) were assessed at six time points (08:00, 12:00, 16:00, 20:00, 24:00, and 04:00).

Assay methodologies and reliability have been previously described [4].

### Statistical analysis

Data are presented as mean ± standard error of the mean (SEM). For descriptive purposes, 90% confidence intervals (IC90) were calculated for each additional parameter and group based on the group mean, standard deviation, and sample size, and are reported in Table 1 alongside the mean ± standard deviation. These IC90 intervals provide an estimation of the precision of group-level values and complement the SEM values reported in the figures and statistical tests. Given the sample size and distribution assumptions, non-parametric tests were used. One-point parameters were compared using Kruskal-Wallis tests, followed by Steel–Dwass post-hoc tests when appropriate. Correlations were assessed using Spearman’s rank correlation.

For repeated measures (3-MT, NM, M), two-way repeated-measures ANOVA (group × time) was performed, followed by appropriate post-hoc comparisons.

No correction for multiple testing was applied given the exploratory nature of the study; therefore, results should be interpreted cautiously. Although non-parametric methods were used for single time-point comparisons, repeated-measures ANOVA was applied for longitudinal data; this approach assumes approximate normality of residuals and should be interpreted accordingly.

**24-hour rhythmicity** of 3-MT, NM and M was assessed using cosinor regression to determine mean level, amplitude and acrophase of the considered parameter with SigmaPlot software (Systat software Inc., San Jose, CA, USA) [28, 29]. Individual data of each studied group (AN, AN-Rec, Controls and CT) were fitted to the following regression: [y = a + b·cos(2·π·(x − c)/24)] where a is the mean level, b the amplitude, and c the acrophase of the rhythm. For a given parameter, mean, size, standard error (SEM) format of SigmaPlot were used to compare the amplitudes and acrophases of significant regressions with One-Way ANOVA followed by Fisher’s post-hoc test for two groups.

Significance level set at 0.05. All analyses were conducted using STATVIEW software (Version 5.0; Abacus Concepts, Inc., Berkeley, CA) and GraphPad Prism 5 Software.

## Results

### General characteristics of the studied population (Table 1)

The four groups were comparable in age.

**Table 1:** General characteristics of the groups of the study: Anthropometric, hormonal and psychometric assessments in the actively ill patients with anorexia nervosa (AN), the patients with ongoing recovery from anorexia nervosa (AN-Rec), the control individuals (Controls), and the patients with Constitutional Thinness (CT). Data are expressed as mean values ± SEM. Resting Energy Expenditure (REE), Dutch Eating Behavior Questionnaire (DEBQ), Eating Disorders Inventory 2 (EDI-2), and Eating Disorders Examination (EDE)

Parameters	AN *N* = 10	AN-Rec *N* = 11	Controls *N* = 10	CT *N* = 10	AN vs. C *P*-value	ANRec vs. C *P*-value	AN vs. ANRec *P*-value	AN vs. CT *P*-value	ANRec vs. CT *P*-value	CT vs. C *P*-value
**Age (years)**	25.4 ± 3.6	25.2 ± 3.7	23.7 ± 1.6	28.3 ± 3.4	0.6674	0.7207	0.9780	0.2735	0.2610	0.1253
**Body Mass Index (kg/m** ^**2**^ **)**	14.3 ± 0.5	17.7 ± 0.9	21.3 ± 0.6	15.2 ± 0.3	**< 0.0001**	**0.0030**	**0.0028**	0.2917	**0.0101**	**< 0.0001**
**Fat mass (%)**	16.5 ± 2.0	21.9 ± 1.9	29.3 ± 1.6	17.4 ± 2.7	**0.0006**	**0.0102**	0.1037	0.7775	0.2032	**0.0018**
**REE (Kcal/24h)**	694 ± 67	835 ± 100	1281 ± 56	933 ± 73	**< 0.0001**	**0.0011**	0.4978	**0.0574**	0.4211	**0.0010**
**Hemoglobin (g/dl)**	12.5 ± 0.5	12.6 ± 0.5	12.9 ± 0.2	13.1 ± 0.5	0.5223	0.6356	0.8856	0.3924	0.4599	0.6983
**Polynuclear neutrophils (x10** ^**9**^ **/l)**	2.1 ± 0.3	2.8 ± 0.4	3.4 ± 0.5	2.8 ± 0.6	**0.0199**	0.3871	0.1778	0.2684	0.9599	0.4234
**Platelets (x10** ^**9**^ **/l)**	171 ± 15	195 ± 15	233 ± 16	222 ± 17	**0.0121**	0.1106	0.2696	**0.0413**	0.2689	0.6430
**Sodium (mmol/l)**	138 ± 0.6	140 ± 06	140 ± 0.5	138 ± 0.6	0.1478	0.5790	0.3709	0.6537	0.3528	0.1302
**Potassium (mmol/l**	4.0 ± 0.1	3.7 ± 0.2	4.0 ± 0.1	3.9 ± 0.1	0.8316	0.2621	0.1877	0.4505	0.3557	0.6833
**Bicarbonates (mmol/l)**	27.5 ± 1.0	27.4 ± 1.4	23.3 ± 0.8	23.7 ± 0.7	**0.0056**	**0.0262**	0.9397	**0.0070**	**0.0361**	0.7195
**Urea (mmol/l)**	5.9 ± 0.8	3.7 ± 0.5	4.9 ± 0.4	4.9 ± 0.3	0.3087	**0.0560**	**0.0310**	0.2831	**0.0647**	0.9194
**Fasting blood glucose (mmol/l)**	3.9 ± 0.1	4.55 ± 0.3	4.6 ± 0.2	4.7 ± 0.1	**0.0234**	0.9510	0.1319	**0.0008**	0.6472	0.5449
**Vitamin B12 (ng/l)**	735 ± 110	445 ± 55	370 ± 31	399 ± 40	**0.0081**	0.2996	**0.0249**	**0.0054**	0.5108	0.6071
**Vitamin B9 (µg/l)**	8.7 ± 0.7	7.9 ± 1.4	4.7 ± 0.5	5.2 ± 0.9	**0.0010**	0.0802	0.6679	**0.0139**	0.1245	0.6376
**Aspartate transaminase (AST) (UI/l)**	139 ± 97	24 ± 1.4	22.4 ± 1.8	21.9 ± 2.1	0.2698	0.3569	0.2284	0.2413	0.2887	0.8447
**Alanine transaminase (ALT) (UI/l)**	116 ± 45	22 ± 2.5	16.0 ± 4.1	16.7 ± 2.2	**0.0558**	0.2109	**0.0448**	**0.0446**	0.1281	0.8801
**Gammaglutamyltransferase (GGT) (UI/l)**	47 ± 16	20 ± 6	15 ± 2	14 ± 1	0.0929	0.4577	0.1381	0.0688	0.3577	0.7161
**Alkaline Phosphate (ALP) (UI/l)**	53 ± 8	42 ± 2	54 ± 3	64 ± 9	0.8734	**0.0035**	0.2127	0.3823	**0.0279**	0.3561
**Albumin (g/L)**	46.3.0 ± 1.9	45.9 ± 2.6	42.9 ± 2.7	43.9 ± 0.5	0.3203	0.4642	0.8808	0.2178	0.4510	0.7172
**Free T**_**3**_ **(pmol/L)**	2.4 ± 0.4	3.9 ± 0.3	5.4 ± 0.3	5.0 ± 0.2	**< 0.0001**	**0.0011**	**0.0089**	**0.0001**	**0.0230**	0.3430
**IGF-I (µg/L)**	118 ± 41	169 ± 24	250 ± 31	251 ± 337	0.0221	**0.0567**	0.2931	0.0669	0.1930	0.7233
**Estradiol (ng/L)**	18.7 ± 10.7	20.8 ± 5.8	102.7 ± 24.1	89.8 ± 33.0	**0.0064**	**0.0026**	0.8650	0.1447	0.1216	0.3880
**LH (UI/L)**	1.2 ± 0.1	12.5 ± 6.9	6.4 ± 1.0	7.1 ± 1.0	**0.0012**	0.3926	0.1420	**0.0004**	0.4704	0.6303
**FSH (UI/L)**	2.2 ± 1.2	22.3 ± 12.5	4.7 ± 0.5	5.3 ± 0.9	0.1244	0.1786	0.1603	0.1076	0.2183	0.5705
**24-hour mean Cross Laps (pmol/L)**	8287 ± 1180	3289 ± 464	3566 ± 354	3732 ± 724	**< 0.0001**	0.6368	**< 0.0001**	**0.0037**	0.3469	0.4872
**24-hour mean Osteocalcin (µg/L)**	9.5 ± 2.0	14.2 ± 0.7	21.9 ± 0.8	25.1 ± 1.3	**< 0.0001**	**< 0.0001**	**0.0089**	**0.0007**	**0.0003**	0.2987
**24-hour mean Leptin (ng/mL)**	2.3 ± 0.4	3.4 ± 0.3	5.4 ± 1.1	3.1 ± 0.4	**0.0243**	**0.0417**	**0.0521**	0.3994	0.1951	**0.0057**
**24-hour mean GH (mUI/L)**	12.7 ± 4.1	4.3 ± 0.5	5.3 ± 0.8	6.7 ± 1.9	0.0670	0.3378	0.0414	0.1124	0.3433	0.6253
**24-hour mean ACTH (ng/L)**	14.1 ± 1.5	17.2 ± 2.0	12.7 ± 1.8	24.3 ± 6.4	0.5567	0.0971	0.2848	0.2232	0.2860	0.0964
**24-hour mean Cortisol (nmol/L)**	399 ± 29	316 ± 29	278 ± 32	264 ± 29	**0.0105**	0.3828	0.0623	**0.0041**	0.2341	0.8130
**24-hour mean Prolactin (mUI/L)**	198 ± 26	375 ± 69	338 ± 31	462 ± 30	**0.0002**	0.6143	**0.0017**	**0.0519**	0.3690	0.3690
**DEBQ - Restrained eating score**	33.7 ± 4.3	29.2 ± 8.7	20.2 ± 3.5	22.0 ± 5.3	**0.0443**	**0.0511**	**0.0911**	**0.0065**	**0.0588**	0.1538
**EDI-2 - Drive for thinness score**	4.0 ± 3.7	2.0 ± 1.8	0.7 ± 0.7	0.3 ± 0.3	**< 0.0001**	**0.0513**	**0.0054**	**< 0.0001**	**0.0003**	0.5385
**EDI-2 - Body dissatisfaction score**	14.6 ± 3.3	14.3 ± 6.6	0.7 ± 0.3	6.0 ± 3.4	**0.0187**	**0.0139**	0.9609	**0.0449**	**0.0314**	**0.0020**
**EDE-Q score**	16.6 ± 2.5	13.8 ± 4.2	1.7 ± 0.8	7.5 ± 3.3	**0.0043**	**0.0755**	0.5818	**0.0583**	0.2968	0.2025

Patients with **acute anorexia nervosa (AN)** displayed significantly lower values for BMI, fat mass, and resting energy expenditure compared to controls. They also showed marked reductions in several metabolic and hormonal parameters, including neutrophil count, platelet count, fasting glucose, free T3, IGF-1, estradiol, LH, FSH, osteocalcin, and leptin. Conversely, they exhibited higher levels of ALT, AST, vitamin B12, vitamin B9, GH, and mean 24-hour cortisol.

Patients with **AN in ongoing recovery (AN-Rec)** showed intermediate values for most metabolic parameters. Their BMI, fat mass, resting energy expenditure, IGF-1, free T3, leptin, and estradiol levels were still significantly lower than those of controls but higher than those of acute AN patients.

**Patients with constitutional thinness (CT)** presented anthropometric and metabolic features similar to controls, with the exception of significantly lower leptin levels.

Psychometric assessment revealed higher scores in both acute AN and AN-Rec compared to CT and controls, consistent with persistent eating disorder psychopathology.

### Mean 24-hour levels of plasma free 3-MT, normetanephrine, and metanephrine (Fig. 1)

**Fig. 1:**
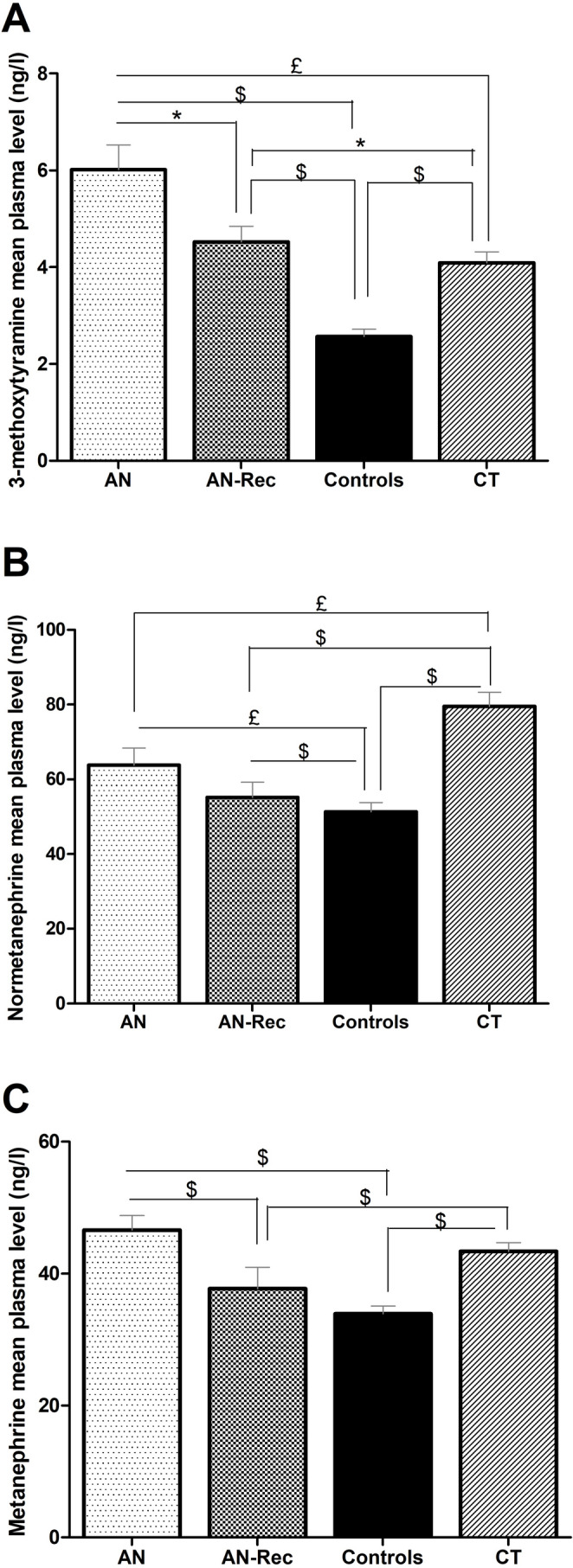
Mean (**A**) 3-methoxytyramine (3MT), (**B**) normetanephrine, and (**C**) metanephrine plasma levels in the groups of the study: the actively ill patients with anorexia nervosa (AN), the ongoing recovery patients with anorexia nervosa (AN-Rec), the control individuals, and the patients with constitutional thinness (CT). Statistics: *: *p* < 0.05 ; £:*p* < 0.01; $:*p* < 0.001

Plasma **3-methoxytyramine (3-MT)** levels over 24 h were significantly higher in acute AN than in all other groups. Intermediate values were observed in AN-Rec and CT, while controls had the lowest levels **(Figs. ****1**** A).**

For **normetanephrine (NM)**, the highest 24-hour mean levels were observed in CT, followed by acute AN. Both groups showed significantly higher levels than controls. AN-Rec displayed values comparable to controls **(Fig. ****1****B).**

**Metanephrine (M)** levels were significantly increased in acute AN and CT compared to both controls and AN-Rec **(Fig. ****1****C).**

### 24-hour profile of plasma free metanephrines (Fig. 2 and Supplemental data)

**Fig. 2:**
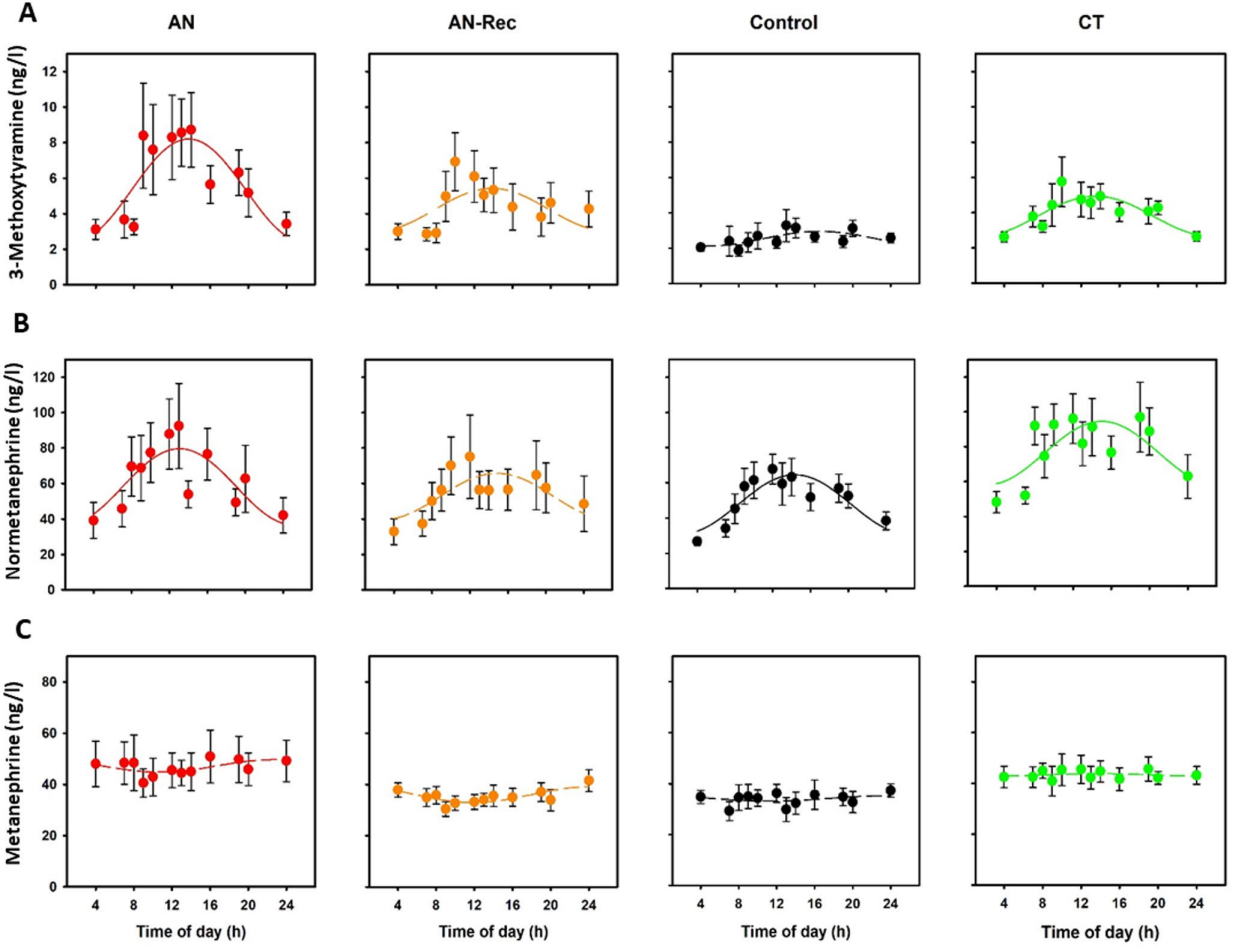
Cosinor regression analysis of 24-hour variations of 3-methoxytyramine (**A**) normetanephrine (**B**), and metanephrine (**C**) plasma levels in the groups of the study: the actively ill patients with anorexia nervosa (AN), the ongoing recovery patients with anorexia nervosa (AN-Rec), the control individuals, and the patients with constitutional thinness (CT). *p* < 0.0001 – fitted solid line; *p* = 0.06 – long-fitted dashed line; non-significant level – fitted short-dashed line

**Cosinor analysis** revealed a clear **rhythmic variation over 24 h** for **3-MT** in each patient group, with similar acrophases close to midday (~ 13h30) and lower levels during the night. Cosinor analysis confirmed the mean levels were higher in AN group (5.33 ± 0.53 ng/L) than in AN-Rec (4.24 ± 0.35 ng/L) or CT group (3.80 ± 0.24ng/L). While the acrophase was not significantly modified according to the patient group, cosinor analysis detected a trend (*P* = 0.068) for higher amplitude in AN group (2.88 ± 0.77 ng/L) compared to AN-Rec and CT group (1.19 ± 0.51 and 1.13 ± 0.35 ng/L, respectively). Detailed post-hoc analysis indicated that patients with AN presented an early peak at 9h00 and maintained a high plateau until 14h00. Both ongoing recovery patients (AN-Rec) and patients with CT presented a later peak at 10h00, followed by a slow decline. The control group did not exhibit significant 24-hour variations **(Fig. ****2****A).**

Cosinor regression analysis of **normetanephrine** 24-hour variations showed significant rhythmicity within all the groups (trend for significance in AN-Rec), with similar acrophase around the midday (~ 13h00). The highest mean values and amplitudes were noticed in CT (76.3 ± 4.01 and 18.2 ± 5.79 ng/L, respectively), followed by AN (58.1 ± 4.7 and 21.4 ± 6.9 ng/L, respectively), AN-Rec (52.7 ± 4.3 and 12.9 ± 6.2 ng/L, respectively) and Controls (47.8 ± 2.5 and 16.7 ± 3.7 ng/L, respectively). Detailed post-hoc analysis indicated patients with CT presented with the earlier and higher peak at 09h00, followed by actively ill patients with AN. Ongoing recovery patients with AN and control individuals presented a smoother peak at 12h00 **(Fig.****2****B).**

Cosinor regression analysis of **metanephrine** did not identify circadian patterns in any of the studied groups **(Fig. ****2****C).**

### Correlations between free metanephrine levels and other parameters

**Across the total sample**,** mean 24-hour 3-MT** correlated positively with vitamin B12 (0.64, *p* < 0.0001), ALT (0.40, *p* = 0.008) and negatively with BMI (-0.45, *p* = 0.0004), platelet count (-0.43, *p* = 0.007) and IGF1 (-0.38, *p* = 0.01). These correlations were also noticed for mean day 3-MT values, delta day/night 3-MT values but not for mean night 3-MT values. **Mean 24-hour NM** values correlated positively with AST (0.37, *p* = 0.01) and negatively with sodium (-0.47, *p* = 0.001). **Mean 24-hour M** value also correlated positively with ALT (0.47, *p* = 0.001) and negatively with BMI (-0.36, *p* = 0.01).

**Strong intercorrelations were found between mean 24-hour 3-MT and metanephrine** (*r* = 0.55, *p* = 0.0004), with weaker correlations between 3-MT and NM (*r* = 0.33, *p* = 0.03), and between NM and M (*r* = 0.38, *p* = 0.01).

No significant correlations were found between metanephrine levels and psychometric scores.

## Discussion

This study is the first to investigate the **24-hour profiles of free plasma metanephrines**, 3-methoxytyramine (3-MT), normetanephrine (NM), and metanephrine (M), in women with **acute anorexia nervosa (AN)**, women in **nutritional recovery from AN (AN-Rec)**, **constitutional thinness (CT)**, and **healthy controls**. Although exploratory in nature, this study provides new insights into catecholamine metabolism in states of low body weight of different etiologies.

The primary finding of the present study is the elevated mean 24-hour plasma levels of free 3-MT, NM and M observed in both patients with acute anorexia nervosa and constitutional thinness compared to healthy participants. These high plasma levels of free metanephrines confirm the results obtained with plasma assays of total metanephrines in malnourished severe anorexia nervosa [1].

A second finding is the identification of a 24-hour variation consistent with a circadian pattern, wherein 3-MT and NM levels are elevated during the daylight hours, predominantly observed in patients exhibiting a low BMI. Such nycthemeral variation may influence the interpretation of single-point assays. However, further studies are required before recommending multi-time-point sampling, in clinical practice, to enhance precision in evaluating the level of these metabolites. Lastly, the presence of this 24-hour rhythm, characterized by significant variations over a 24-hour cycle, in patients with acute anorexia nervosa, may explain the contradictory nature of existing literature data on catecholamines levels in the context of anorexia nervosa.

The high mean 24-hour levels of 3-MT in patients with anorexia nervosa in the acute phase may underlie an increased dopaminergic activity and further raise questions about the triggering mechanisms. Firstly, a mechanism related to undernutrition should be considered, likely largely due to the chronic hypoglycaemia characterizing these patients [2], a factor which was unfortunately not investigated in this study. The hypothesis of a nutritional determinism for these catecholaminergic abnormalities, and therefore of their reversibility in anorexia nervosa, is also suggested by the presence of decreasing levels of 3-MT, metanephrine and normetanephrine in patients with AN while regaining weight when compared to those in acute phase. Patients in the acute phase of anorexia nervosa were previously found with decreased prolactin levels [30, 31], results which were not confirmed in the present study. However, it is well known that dopamine, the precursor of 3-MT, is the main inhibitor of prolactin synthesis. Therefore, the lower prolactin release may be linked to this enhanced dopaminergic activity during the acute phase of undernutrition in anorexia nervosa. Interestingly, in activity-based anorexia (ABA) animal undernourished models, an increase in dopamine and dopamine receptors density levels was observed in several areas of the reward circuit [32]. In a previous study, we also showed an activation of opioid system in key cerebral regions of the reward circuit in the undernourished patients with anorexia nervosa [33]. All these corroborated elements suggest that this acute phase of undernutrition can create a neuromediated state of dependence [34, 35] which makes it more difficult for patients to decide to stop restricting and resume eating to regain weight [36, 37]. These hypotheses remain speculative and were not directly assessed in the present study.

Besides the potential neuromodulatory role in eating behaviour, the rise of catecholamine and metabolite levels may also have a potential negative collateral impact on the metabolism. These abnormalities may be involved in the paradoxical increase in metabolic rate described in some severe undernourished patients anticipating death [38]. They also may represent a potential hidden risk for cardiac rhythm disorders in people considered, according to symptomatology (bradycardia) and ECG Holter recordings of autonomous nervous system, to be rather under the predominant influence of a parasympathetic tone [39, 40]. Ultimately, this most often clinically overlooked increase in catecholamines may theoretically contribute to cardiovascular risk, although not assessed in the present study [3]. Lately it was shown that 3-MT, considered as a trace amine, could also act as a neuromediator through specific receptors (TAAR1) [12, 13] to induce a resistance to weight gain [14]. It would be interesting to develop further studies focused on this interplay in anorexia nervosa, especially in chronic forms.

Several metabolic and nutritional aspects linked to circadian rhythm could explain the greater diurnal vs. nocturnal activation of normetanephrine and 3-MT synthesis: diurnal activation of the digestive tract, and presence of meals or physical activity. In our study, this diurnal activation in patients with acute anorexia nervosa seems to be enhanced by both energy and liver exhaustion, suggested by correlations with abnormalities of biological parameters which synthesize, store or control hepatocyte functions: decreased IGF-1, increased ALT, and, especially, vitamin B12. The increase of cobalamin / vitamin B12 in anorexia nervosa has been, recently, highlighted in the literature as a potential marker of disease severity [41, 42]. Corroborated with all these indicators of severity, the assessment of metanephrines could be explored in future studies as potential markers, but their role in clinical management remains to be established.

One result considered surprising is the higher levels of 3-MT, normetanephrine and metanephrine in the constitutionally thin participants than in control subjects. While in anorexia nervosa, undernutrition may explain the activation of catecholamine synthesis, no metabolic, nutritional or hormonal elements indicating a state of undernutrition can be found in patients with constitutional thinness [4]. Therefore, the higher levels of catecholamines observed in CT could reflect underlying biological traits, possibly reflecting constitutive traits associated with satiety regulation [43], and a possible stimulation of brown fat activity [6, 44], although this remains speculative and requires dedicated mechanistic studies. Genetic evaluation of catecholamines metabolism in this lean category could address this hypothesis. An excess of protein intake has been demonstrated in patients with CT relative to their low bodyweight and lean mass, associated with higher urinary excretion of essential amino acids [5]. It is well known that protein intake preferentially stimulates postprandial thermogenesis and it was shown in animal models that hypothalamic norepinephrine is also increased during protein intake [45]. It is therefore also possible that this overall diurnal increase in catecholamines metabolites in constitutional thinness would be linked to this relatively high protein intake. This remains to be demonstrated in controlled studies using standardized test meals.

This study has several limitations. First, the **sample size was modest**, and no formal power calculation was performed, which may limit the ability to detect subtle differences, particularly in the analysis of 24-hour variations. However, the intensive design, with 12 time points over 24 h per participant, allowed detailed intra-individual profiling and robust 24-hour modeling despite the limited number of subjects. Second, multiple statistical comparisons were performed without correction, increasing the risk of type I error. As such, the results should be interpreted with caution and considered hypothesis-generating. Third, the controlled in-patient setting, including standardized meals and restricted activity, may not fully reflect real-life conditions and could have influenced the observed temporal patterns. Fourth, plasma metanephrines reflect peripheral catecholamine metabolism and do not directly inform central catecholaminergic activity. Finally, several potentially relevant physiological factors, including continuous glycemic fluctuations, sleep quality, and prior physical activity, were not assessed and may have influenced the observed results. Taken together, these limitations emphasize the exploratory nature of the present study and the need for confirmation in larger and independent cohorts.

**To conclude**, this exploratory study suggests that free plasma metanephrine levels and their 24-hour variation differ between anorexia nervosa, constitutional thinness, and healthy states. Elevated levels observed in both conditions of low body weight may reflect distinct underlying physiological mechanisms and are associated with markers of metabolic and nutritional status.

These findings highlight the importance of temporal variation in interpreting metanephrine levels. However, their physiological significance remains to be clarified. Given the limited sample size and exploratory design, further studies in larger and more diverse populations are required to confirm these findings and better define their clinical and physiological relevance.

## Supplementary Information

Below is the link to the electronic supplementary material.

Supplementary Material 1

## Data Availability

The data that support the findings of this study are available from the corresponding author upon reasonable request.
