# Supplementary material for: Circadian profiles of free plasma metanephrines in anorexia nervosa and constitutional thinness
Source: Endocrine. 2026 Jun 8;91(1):207. doi: 10.1007/s12020-026-04648-x (PMC13246810; doi:10.1007/s12020-026-04648-x)
Supplement: Supplementary file 1 — Supplementary Material 1 [file 12020_2026_4648_MOESM1_ESM.docx]

| **3-Methoxytyramine** | | | | | | |
| --- | --- | --- | --- | --- | --- | --- |
| **Parameter** | **Group** | **Mean** | **IC 95%** | **SEM** | **N** | **P (LSD post hoc)** |
| MESOR | AN | 5.33 | 4.13 – 6.53 | 0.53 | 10 | p=0.032 vs AN-Rec; p<0.001 vs. Controls; p=0.004 vs. CT |
| MESOR | AN-Rec | 4.24 | 3.46 – 5.02 | 0.35 | 11 | p=0.001 vs. CT |
| MESOR | Controls | 2.54 | 2.16 – 2.92 | 0.17 | 10 |  |
| MESOR | CT | 3.81 | 3.27 – 4.35 | 0.24 | 10 | p=0.016 vs. Controls |
| **P global ANOVA** : <0.001 | | | | | | |
| AMPLITUDE | AN | 2.88 | 1.12 – 4.64 | 0.78 | 10 |  |
| AMPLITUDE | AN-Rec | 1.19 | 0.05 – 2.33 | 0.51 | 11 |  |
| AMPLITUDE | CT | 1.13 | 0.34 – 1.92 | 0.35 | 10 |  |
| **P global ANOVA** : 0.068 | | | | | | |
| ACROPHASE | AN | 13.75 | 11.71 – 15.79 | 0.9 | 10 |  |
| ACROPHASE | AN-Rec | 13.83 | 10.64 – 17.02 | 1.43 | 11 |  |
| ACROPHASE | CT | 13.49 | 11.14 – 15.84 | 1.04 | 10 |  |
| **P global ANOVA** : 0.977 | | | | | | |
| **Normetanephrine** | | | | | | |
| MESOR | AN | 58.16 | 47.32 – 69.00 | 4.79 | 10 | p=0.003 vs. CT |
| MESOR | AN-Rec | 52.77 | 43.14 – 62.40 | 4.32 | 11 | p<0.001 vs. CT |
| MESOR | Controls | 47.81 | 42.00 – 53.62 | 2.57 | 10 | p<0.001 vs. CT |
| MESOR | CT | 76.32 | 67.23 – 85.41 | 4.02 | 10 | p<0.001 vs. All other groups |
| **P global ANOVA** : <0.001 | | | | | | |
| AMPLITUDE | AN | 21.44 | 5.76 – 37.12 | 6.93 | 10 |  |
| AMPLITUDE | AN-Rec | 12.97 | -0.93 – 26.87 | 6.24 | 11 |  |
| AMPLITUDE | Controls | 16.72 | 8.28 – 25.16 | 3.73 | 10 |  |
| AMPLITUDE | CT | 18.23 | 5.13 – 31.33 | 5.79 | 10 |  |
| **P global ANOVA** : 0.773 | | | | | | |
| ACROPHASE | AN | 13.06 | 10.59 – 15.53 | 1.09 | 10 |  |
| ACROPHASE | AN-Rec | 14.83 | 11.20 – 18.46 | 1.63 | 11 |  |
| ACROPHASE | Controls | 14.34 | 12.67 – 16.01 | 0.74 | 10 |  |
| ACROPHASE | CT | 15.01 | 12.57 – 17.45 | 1.08 | 10 |  |
| **P global ANOVA** : 0.669 | | | | | | |
| **Metanephrines** | | | | | | |
| MESOR | AN | 47.25 | 40.9 0- 52.03 | 2.44 | 10 |  |
| MESOR | AN-Rec | 36.03 | 34.54 - 37.51 | 1.06 | 11 |  |
| MESOR | Controls | 34.23 | 32.41 - 36.05 | 1.75 | 10 |  |
| MESOR | CT | 43.30 | 42.01 - 44.60 | 1.38 | 10 |  |
| **P global ANOVA :**0.1665 | | | | | | |
| AMPLITUDE | AN | 3.05 | -0.25-5.10 | 3.25 | 10 |  |
| AMPLITUDE | AN-Rec | 4.64 | 0.95-5.14 | 1.36 | 11 |  |
| AMPLITUDE | Controls | 1.81 | -1.48-3.55 | 1.67 | 10 |  |
| AMPLITUDE | CT | -0.528 | -2.41-1.35 | 1.64 | 10 |  |
| **P global ANOVA :** 0.8132 | | | | | | |
| ACROPHASE | AN | -1.348 | -5.63-2.93 | 2.46 | 10 |  |
| ACROPHASE | AN-Rec | -0.104 | -2.55-2.34 | 1.18 | 11 |  |
| ACROPHASE | Controls | -0.716 | -9.73-8.30 | 1.10 | 10 |  |
| ACROPHASE | CT | 1.661 | -10.24-13.57 | 2.17 | 10 |  |
| **P global ANOVA : 0.6859** | | | | | | |
